# Longitudinal strain correlates with 6-minute walk distance whereas ejection fraction and diastolic parameters do not

**DOI:** 10.1186/s12947-024-00325-z

**Published:** 2024-06-07

**Authors:** John W. Petersen, Natalie Bracewell, Kevin M. Schneider, Joshua Latner, Shuang Yang, Yi Guo

**Affiliations:** 1 Division of Cardiovascular Medicine, University of Florida, 1600 SW Archer Road, PO Box 100288, Gainesville, FL 32610 USA; 2 Department of Internal Medicine, University of Florida, Gainesville, FL USA; 3 Department of Health Outcomes and Biomedical Informatics, University of Florida,, Gainesville, FL USA

## Abstract

**Background:**

Impaired functional capacity is a common symptom in patients with heart failure. Standard measures of left ventricular (LV) function, such as ejection fraction (EF) and LV diastolic parameters, do not correlate with measures of functional capacity. The aim of this study is to determine if measures of global and regional LV strain better correlate with 6-minute walk distance than does EF or measures of LV diastolic function.

**Methods:**

120 patients referred to a cardiology clinic for evaluation of known or suspected heart failure were approached for enrollment. Of those 120 patients, 58 had an echocardiogram within 3 months of enrollment with images adequate for regional and global strain assessment, had no contra-indication to exercise testing, and had no previously documented non-cardiac explanation for dyspnea on exertion. In those 58 patients, 6-minute walk distance was measured, LV EF was determined with Simpson’s biplane method, and global and regional longitudinal strain were measured with TomTec Image Arena 4.5.1 software.

**Results:**

LV EF had no correlation with 6-minute walk distance (r = 0.22, p = 0.09) even when controlling for age, gender, and BMI (p = 0.07). No measures of LV diastolic function (including E velocity, Deceleration Time, e’ annular velocities, or E/e’) had a correlation with 6-minute walk distance. Multiple measures of global and regional LV longitudinal systolic function had a correlation with 6-minute walk distance. Longitudinal strain of the basal LV segments had the strongest correlation with 6-minute walk distance (*r*= -0.36, *p* = 0.005), and correlation persisted after controlling for age, gender, BMI, and systolic blood pressure (*p* = 0.004).

**Conclusions:**

Longitudinal strain correlates with a measure of functional capacity, but LVEF and traditional measures of LV diastolic dysfunction do not. Measures of longitudinal strain, especially in basal LV segments, will likely be an important marker of clinically relevant LV function.

**Graphical Abstract:**

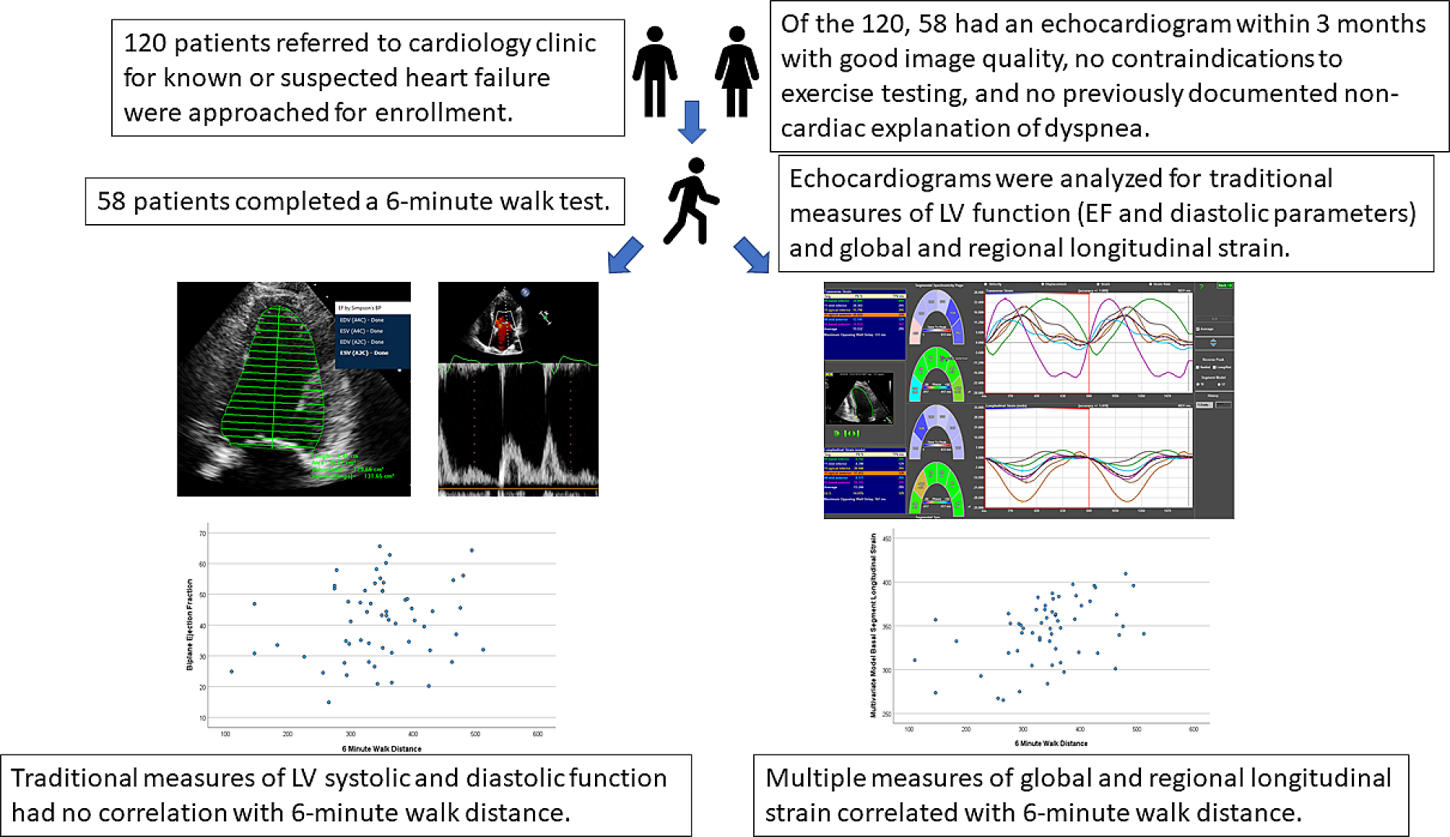

## Background

Impaired functional capacity provides significant disability and signals poor prognosis in patients with heart failure [1]. Clinicians often aim to improve both mortality and impaired functional capacity by improving left ventricular (LV) function with medications, revascularization, and cardiac resynchronization procedures. LV function is most often estimated with measures of ejection fraction (EF) [2]. Unfortunately, EF does not provide an appropriate target for improved functional capacity as it does not correlate with quantitative measures of functional capacity [3–6]. Further, Bhatia et al. demonstrated that 31% of patients hospitalized with heart failure had a preserved LV EF, and mortality and readmission rates were not different between those with preserved or reduced LV EF [7]. We aim to identify a measure of LV function that better correlates with functional capacity and could serve as an appropriate therapeutic target.

 We previously demonstrated that speckle tracking echo-determined measures of LV global and regional longitudinal contraction had a strong linear association with estimates of functional capacity [8]. The measure of functional capacity in that study was the Duke Activity Status Index (DASI) questionnaire. While DASI is a convenient measure of functional capacity that has shown important prediction of outcomes in patients with heart failure, some studies have shown poor correlation of DASI with maximal oxygen consumption [9]. In this study, we aimed to determine if measures of LV global and regional longitudinal contraction would correlate with a direct measure of functional capacity. 6-minute walk distance is known to be a strong predictor of outcomes in patients with heart failure [10]. Therefore, we aimed to correlate measures of LV global and regional longitudinal contraction with 6-minute walk distance.

## Methods

### Patients

This study was approved by the University of Florida institutional review board (IRB). Adult patients referred to the University of Florida for evaluation of heart failure or LV dysfunction who also had a clinically indicated echocardiogram within the prior three months were approached for enrollment. Patients with moderate or severe valve disease or echo images inadequate for speckle tracking assessment of LV longitudinal strain were not included in the study. Patients with a contraindication to exercise according to the ACC/AHA guidelines for exercise testing [11] were not included. Patients with a previously documented non-cardiac explanation for dyspnea on exertion were not included.

### Echocardiography

Echocardiograms were obtained with an iE33 (Philips, The Netherlands). Apical 2 and 4 chamber images were used to measure LV EF by Simpson’s biplane method. Pulsed wave Doppler at the level of the mitral valve leaflet tips was used to determine peak early (E) and atrial (A) filling velocities and deceleration time (DT). Tissue Doppler was used to determine peak early (e’) velocity of the medial and lateral mitral annulus.

### Speckle tracking analysis

Analysis was performed using TomTec software (Image Arena Version 4.6, Germany). Longitudinal strain was measured for each of 6 segments in the apical 4, 2, and 3 chamber images (18 segments total). Tracking quality of each segment was visually evaluated, and if tracking was felt to be inaccurate, strain analysis for that segment was not included. Longitudinal strain was determined for each of the six LV walls (anteroseptal, anterior, anterolateral, inferolateral, inferior, and inferoseptal) by averaging the three segments in each of those walls. Longitudinal strain was also determined for each of the three regions of the LV (apical, mid, and basal) by averaging the six segments in each of those regions. Global longitudinal strain was determined by averaging longitudinal strain in all 18 of the LV segments measured with TomTec. If fewer than 2/3 of the variables for a computed variable were felt to have accurate tracking, then that computed variable was not included in the analysis.

### Statistical analysis

Variables were tested for normality by checking for skewness and kurtosis followed by the Kolmogorov–Smirnov test. For variables that did not follow the normal distribution, we performed a log transformation. Correlations between all the variables were assessed using the Pearson’s correlation coefficients. Variables showing high levels of collinearity (correlation coefficients > 0.7) were eliminated from consideration in the regression model. We employed multivariable linear regression models that were adjusted for four covariates: age, gender, BMI, and systolic blood pressure. In our analytical approach, we conducted a series of separate analyses. Initially, we individually incorporated each measurement into the model to assess its singular association with the 6-minute walk distance while controlling for the covariates. This step allowed us to examine the distinct impact of each measurement on the 6-minute walk distance. Following these initial individual analyses, we proceeded with multivariable forward stepwise linear regression. In this phase, we included the covariates as well as the measurements that had demonstrated an association with the 6-minute walk distance in the initial step. The forward stepwise approach involved selecting the most statistically significant variables and sequentially integrating them into the model until we achieved the final model. All statistical analyses were performed using SAS version 9.4 software (Cary, NC). All p-values were two-sided and p-value < 0.05 was considered statistically significant.

## Results

### Patients

120 patients referred to a cardiology clinic for evaluation of known or suspected heart failure were approached for enrollment. Of those 120 patients, 58 had an echocardiogram within 3 months of enrollment with images adequate for regional and global strain assessment, had no contra-indication to exercise testing, and had no previously documented non-cardiac explanation for dyspnea on exertion. Of these 58 patients, 54 already had a diagnosis of cardiomyopathy by a cardiologist at the time of enrollment. The other four patients had suspected cardiomyopathy based on exertional symptoms without a non-cardiac explanation at the time of enrollment. Average 6-minute walk distance for these 58 patients was 344 (± 83) meters, age 57 (± 11.7) years, weight 194 (± 40) lbs, BMI 29 (± 5), systolic blood pressure 123 (± 18) mmHg, diastolic blood pressure 76 (± 11) mmHg, creatinine 1.4 (± 2.2) mg/dL, and hemoglobin 13.2 (± 1.8) mg/dL. 71% of patients were male, and 85% were white. 43% had prior coronary revascularization, and 14% had a history of atrial fibrillation. Cardiac biomarkers were not obtained as part of this research protocol, but 25 subjects had NT-BNP, and 20 subjects had TnT acquired within 30 days of their echocardiogram for clinically indicated reasons. Average NT-BNP was significantly increased at 3647 (± 4748) pg/mL, but there was a wide range (92 − 17,587pg/mL). Average TnT was near normal at 0.34 (± 0.8ng/mL).

### Standard echo measures

**Fig. 1:**
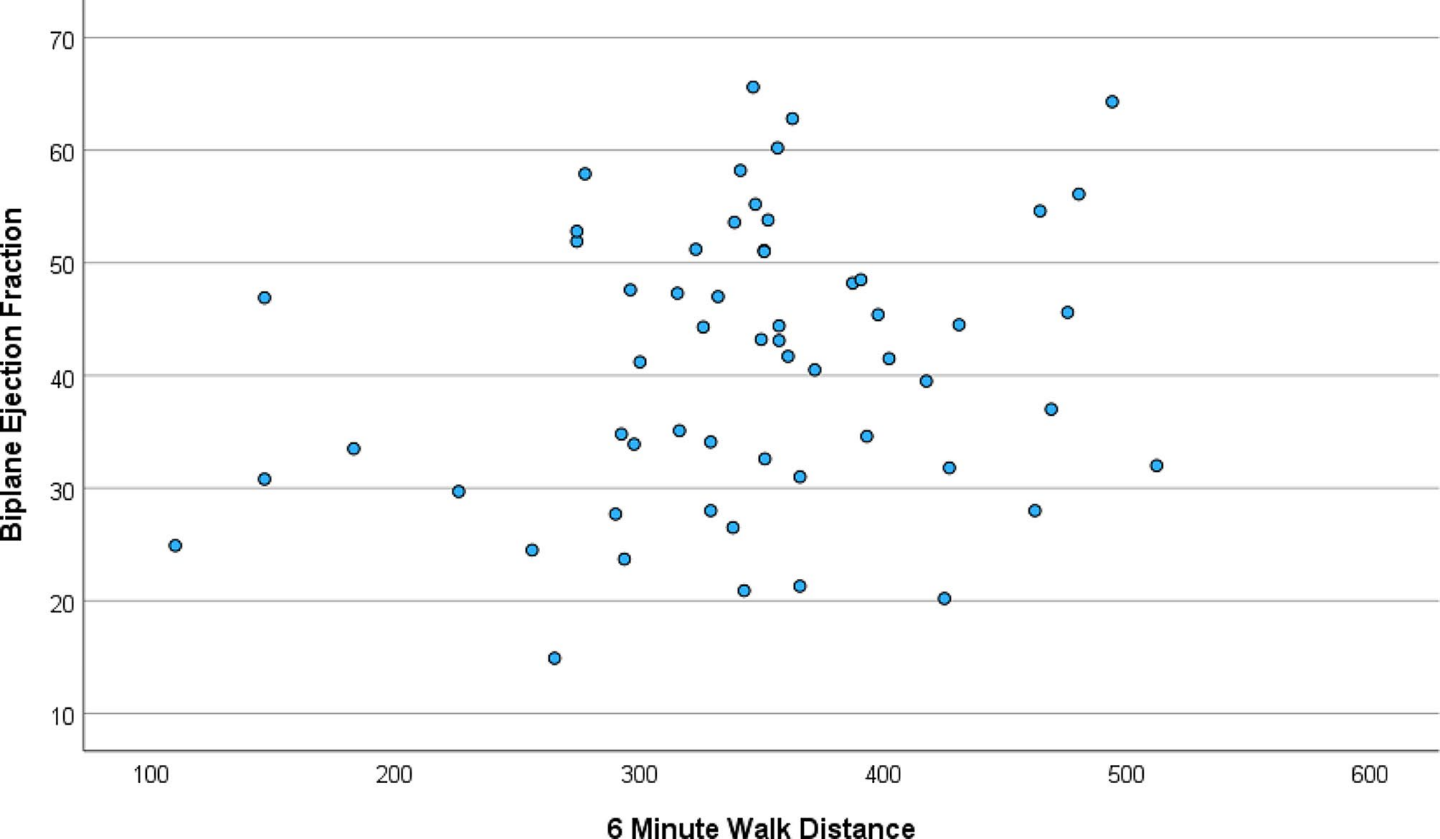
Scatter plot demonstrating correlation of 6-minute walk distance (meters) and Ejection Fraction (%)

Standard echocardiogram measures are listed in Table 1. 16 of the 58 patients (28%) had an EF > 50%. Based on the Kolmogorov–Smirnov test, traditional measures of LV diastolic function were not normally distributed, and we performed a log-normal transformation for these measures. All of the log transformed measures of LV diastolic function and LV EF did not have any correlation with 6-minute walk distance (Table 2; Fig. 1). Further, none of these traditional measures of LV systolic and diastolic function correlated with 6-minute walk distance when adjusted for age, gender, and BMI.

**Table 1:** Standard Echo Measures

	*N*	Mean	Standard Deviation	Maximum	Minimum
**Biplane Ejection Fraction (%)**	58	41.2	12.4	65.6	14.9
**Biplane End Systolic Volume (mL)**	58	87.6	48.6	281.7	26.4
**Biplane End Diastolic Volume (mL)**	58	137.3	55.4	330.9	56.0
**E (cm/s)**	48	81.3	19.6	127	51
**A (cm/s)**	48	64.9	26.2	121	19.7
**E/A**	47	1.5	0.75	3.5	0.48
**Deceleration Time (msec)**	49	216.5	79.8	447	76
**e’ medial (cm/s)**	46	6.3	2.2	12.2	3
**e’ lateral (cm/s)**	44	9.1	3.6	17.6	3.6
**E/e’ medial**	44	14.3	6.3	33.7	7.1
**LV Diastolic Diameter (cm)**	58	5.5	0.78	7.9	3.7
**LV Systolic Diameter (cm)**	58	4.3	0.98	6.9	2.1
**Ventricular Septum Thickness (cm)**	58	0.97	0.17	1.5	0.7
**Posterior Wall Thickness (cm)**	58	0.96	0.18	1.6	0.6
**Left Atrial Diameter (cm)**	58	4.0	0.7	5.6	2.2

**Table 2:** Correlation of traditional measures of LV systolic and diastolic function with 6-minute walk distance

Correlation with 6-minute walk distance	*N*	Correlation Estimate^a^	*p* Value^b^
**Biplane Ejection Fraction**	58	0.21899	0.0986
**log E/e’ medial**	44	-0.18010	0.2420
**log e’ lateral**	44	0.09546	0.5377
**log e’ medial**	46	0.16251	0.2806
**log deceleration time**	49	0.13687	0.3484
**log E**	48	-0.00851	0.9542
**log A**	48	-0.05602	0.7053
**log E/A ratio**	47	0.04471	0.7654

a. The correlation estimate based on log transformation

b. Significance set at *p* < 0.05

### Global and regional left ventricular strain

Many measures of global and regional LV strain correlated with 6-minute walk distance. Global longitudinal strain (*r*= -0.312, *P* = 0.017), longitudinal strain of basal LV segments (*r* = -0.359, *P* = 0.006), longitudinal strain of anterior LV segments (*r* = -0.321, *P* = 0.014), and longitudinal strain of anterolateral LV segments (*r* = -0.292 *P* = 0.026) were negatively correlated with 6-minute walk distance. (Table 3).

**Table 3:** Correlation of measures of global and regional longitudinal strain with 6-minute walk distance

Correlation with 6-minute walk distance	*N*	Correlation Estimate^a^	*p* Value^a^
**Global Long. Strain**	58	-0.312	**0.017**
**Long. Strain Basal LV segments**	58	-0.359	**0.006**
**Long. Strain Mid LV segments**	58	-0.209	0.115
**Long. Strain Apical LV segments**	58	-0.254	0.054
**Long. Strain Anteroseptal LV seg.**	56	-0.202	0.136
**Long. Strain Anterior LV segments**	58	-0.321	**0.014**
**Long. Strain Anterolateral LV seg.**	58	-0.292	**0.026**
**Long. Strain Inferolateral LV seg.**	57	-0.259	0.051
**Long. Strain Inferior LV segments**	58	-0.323	0.013
**Long. Strain Inferoseptal LV seg.**	58	-0.252	0.057

a. Significance set at *p* < 0.05

### Multivariable linear regression analysis

The multivariable linear regression to determine the individual measurement association with 6-minute walk distance shows that after adjusting for age, gender, BMI, and systolic blood pressure, longitudinal strain of basal LV segments (β = -8.28, *P* = 0.004), longitudinal strain of anterior LV segments (β = -6.18, *P* = 0.011), longitudinal strain of anterolateral LV segments (β = -5.91, *P* = 0.018), longitudinal strain of inferoseptal LV segments (β = -4.68, *P* = 0.043), and longitudinal strain of inferolateral LV segments (β = -5.6, *P* = 0.043) were significantly and independently associated with 6-minute walk distance.

**Fig. 2:**
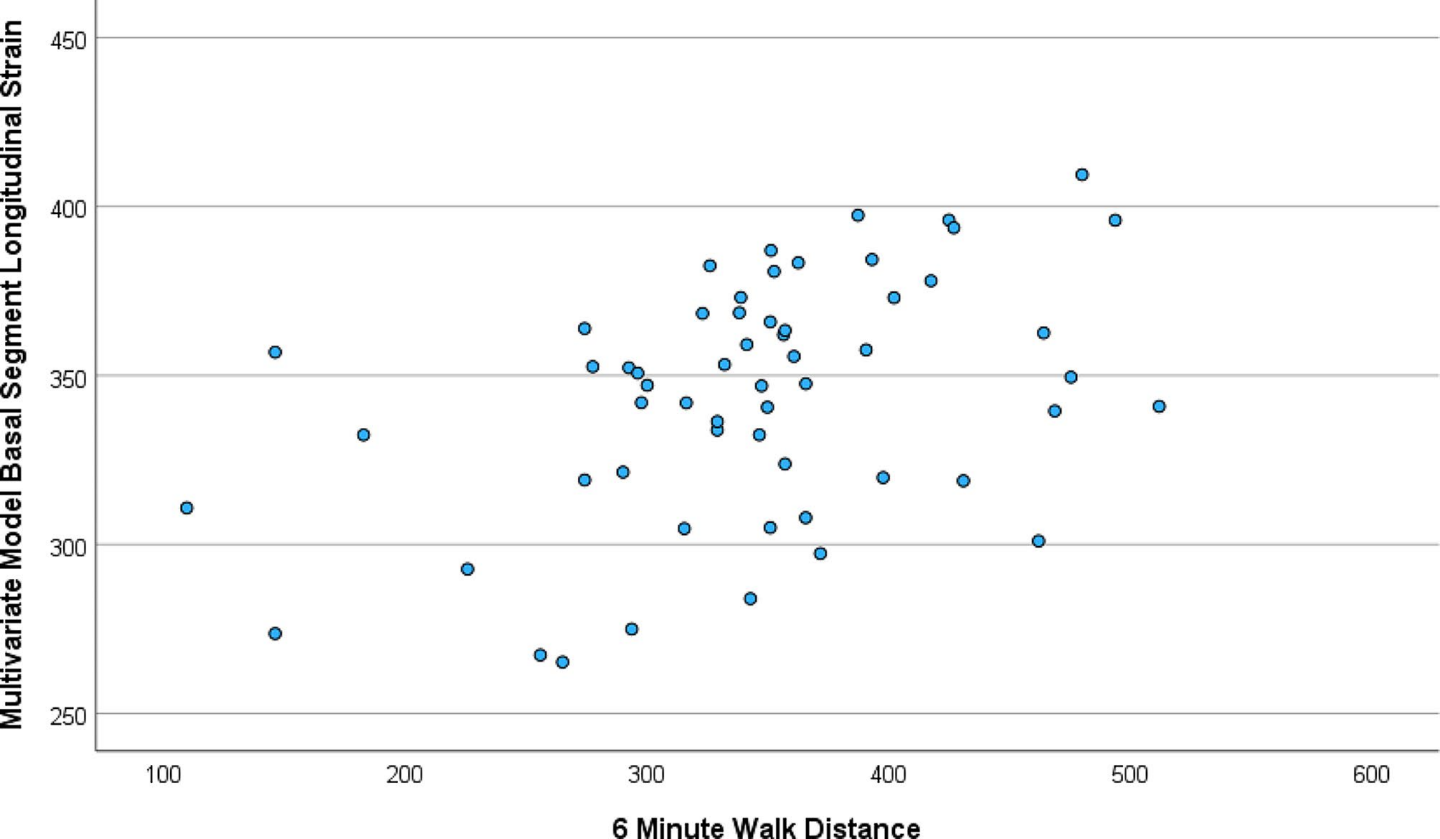
Scatter plot demonstrating correlation of 6-minute walk distance (meters) and the multivariate model using age, gender, BMI, systolic blood pressure and longitudinal strain of basal LV segments

Then we performed multivariable forward stepwise linear regression that adjusted for age, gender, BMI, and individual measurements that were significantly associated with 6-minute walk distance in the above analysis. The final model shows that after controlling for gender (β = -19.0, *P* = 0.411), age (β = -0.61, *P* = 0.496), BMI (β = 0.31, *P* = 0.877), and systolic blood pressure (β = 0.83, *P* = 0.168), longitudinal strain of basal LV segments (β = -8.28, *P* = 0.004) was significantly and independently associated with 6-minute walk distance. Therefore, the final model to predict 6-minute walk distance is: 194-(0.61*age)-(19.0*gender)+(0.31*BMI) +(0.83*SBP) -(8.28*longitudinal strain of basal LV segments). Figure 2 demonstrates scatter plots comparing 6-mintue walk distance and the final multi-variate model.

## Discussion

Reduced functional capacity is a common problem in patients with cardiac disease [12]. Unfortunately, the most common measure of left ventricular function, EF, has previously been shown to not correlate with quantitative measures of functional capacity [3, 4]. Similarly, in the present study we showed no correlation between EF and 6-minute walk distance. Further, standard measures of LV diastolic function did not correlate with 6-minute walk distance. However, measures of LV longitudinal strain, especially longitudinal strain of the basal LV segments, had a significant correlation with 6-minute walk distance.

While our study demonstrated association of basal longitudinal strain with functional capacity in patients without significant valve disease, other studies have shown a correlation of longitudinal strain of the basal LV segments with exercise capacity and clinical outcome in patients with valve disease. Lafitte et al. demonstrated that in patients with severe aortic stenosis, longitudinal strain in basal LV segments was significantly lower in patients with abnormal symptom-limited treadmill testing as compared to those with normal treadmill tests (-11.0 ± 2.0 vs. -14.1 ± 2.4%) [13]. Similarly, Dulgheru et al. evaluated for correlation of measures of regional longitudinal strain with exercise capacity (measured during cardiopulmonary exercise testing) in patients with moderate or severe aortic stenosis and found that longitudinal strain of basal and apical LV segments strongly correlated with peak oxygen consumption (peak VO_2_) [14]. Just as was shown in our study, after multivariable analysis, longitudinal strain of the basal LV segments had the strongest correlation with exercise capacity.

The fact that basal LV segment longitudinal shortening correlates better with clinical outcome than does longitudinal shortening of apical or mid LV segments may relate to the normal timing of LV segmental contraction. Sengupta et al. demonstrated that longitudinal shortening first occurs in the apical and mid LV segments and then in basal LV segments [15, 16]. Deterioration of basal LV segment longitudinal strain may be accentuated by the impact of increased afterload in late-systole. For example, patients with aortic stenosis will have progressive increase in LV pressure during systole with therefore greater stress placed when basal LV segments are contracting [13].

Additionally, during early diastole, it has been shown that a pressure gradient exists between the apex and base of the heart with lower pressure at the apex. This pressure gradient promotes suction of blood from the open mitral valve toward the apex of the left ventricle. In a normal heart, blood flow across the mitral valve that is directed toward the LV apex quickly forms into a vortex [17, 18]. This vortical blood flow promotes efficient redirection of blood from the LV inflow toward the LV outflow tract. It is possible that changes in basal LV segment contractility and relaxation disrupt the normal base to apex intra-ventricular pressure gradient and alter the formation of an efficient intra-ventricular vortex. The orientation of the mitral orifice relative to the left ventricle has also been shown to correlate with efficient vortex formation [19]. When the mitral orifice is too centered to the LV or is too eccentrically oriented to the LV, there is a decrease in efficiency. Changes in basal LV segment contractility could alter mitral orifice orientation that impairs intra-ventricular blood flow efficiency.

There are limitations to our study. We recruited as many patients as our budget would allow, but our sample size was small. Fortunately, we were able to detect significant correlation with novel measures of LV function with 6-minute walk distance despite our small sample size. Echocardiograms used for analysis of this study were performed within 3 months of 6-minute walk testing. Ideally echocardiograms would have been acquired on the same day of 6-minute walk testing, but our budget required use of echocardiograms acquired for clinically indicated reasons. No patient had acute coronary syndrome or decompensated heart failure between time of echocardiogram and 6-minute walk testing, and we are hopeful that use of clinically indicated echocardiograms will allow generalizability to the typical correlation of reported functional capacity in an outpatient clinic to the results of a clinically indicated echocardiogram. Maximal oxygen consumption (MVO2) testing is considered the gold standard for assessment of functional capacity in cardiovascular research [20]. Unfortunately, MVO2 testing can be expensive and less convenient for heart failure patients. 6-minute walk testing is readily available in almost all clinics and has been shown to predict clinical outcomes in outpatients with heart failure, irrespective of etiology [10].

## Conclusions

Measures of LV longitudinal strain, especially in basal LV segments, correlate with measures of functional capacity, while traditional measures of LV systolic and diastolic function do not. Including these measures of LV strain when evaluating patients with heart failure and reduced functional capacity is important. Global and regional longitudinal strain may be an effective therapeutic target for therapies aimed at improving functional capacity.

## Data Availability

Data for this project is not publicly available with hopes of maintaining subject privacy. We are hopeful that scatter plots adequately demonstrate de-identified individual subject data used in our analysis. Requests for the complete dataset will be reviewed and if reasonable data will be provided if approved by University of Florida Institutional Review Board.
