# Correction: Longitudinal strain correlates with 6-minute walk distance whereas ejection fraction and diastolic parameters do not

**DOI:** 10.1186/s12947-024-00327-x

**Published:** 2024-07-05

**Authors:** John W. Petersen, Natalie Bracewell, Kevin M. Schneider, Joshua Latner, Shuang Yang, Yi Guo

**Affiliations:** 1 Division of Cardiovascular Medicine, University of Florida, 1600 SW Archer Road, PO Box 100288, Gainesville, FL 32610 USA; 2 Department of Internal Medicine, University of Florida, Gainesville, FL USA; 3 Department of Health Outcomes and Biomedical Informatics, University of Florida, Gainesville, FL USA

**Correction: Cardiovasc Ultrasound 22, 6 (2024).**

10.1186/s12947-024-00325-z.

Following publication of the original article [1], the authors identified an error in Table 3. For Long. Strain Inferior LV segments, it was published as correlation estimate of -0.035 with p value 0.792. The correct correlation estimate is -0.323 with p value 0.013).

The correct Table 3 is given below.

**Table 3:** Correlation of measures of global and regional longitudinal strain with 6-minute walk distance

Correlation with 6-minute walk distance	*N*	Correlation Estimate^a^	*p* Value^a^
**Global Long. Strain**	58	-0.312	**0.017**
**Long. Strain Basal LV segments**	58	-0.359	**0.006**
**Long. Strain Mid LV segments**	58	-0.209	0.115
**Long. Strain Apical LV segments**	58	-0.254	0.054
**Long. Strain Anteroseptal LV seg.**	56	-0.202	0.136
**Long. Strain Anterior LV segments**	58	-0.321	**0.014**
**Long. Strain Anterolateral LV seg.**	58	-0.292	**0.026**
**Long. Strain Inferolateral LV seg.**	57	-0.259	0.051
**Long. Strain Inferior LV segments**	58	-0.323	**0.013**
**Long. Strain Inferoseptal LV seg.**	58	-0.252	0.057

The original article has been corrected.
